# Novel anti-CD3 chimeric antigen receptor targeting of aggressive T cell malignancies

**DOI:** 10.18632/oncotarget.11019

**Published:** 2016-08-02

**Authors:** Kevin H. Chen, Masayuki Wada, Amelia E. Firor, Kevin G. Pinz, Alexander Jares, Hua Liu, Huda Salman, Marc Golightly, Fengshuo Lan, Xun Jiang, Yupo Ma

**Affiliations:** ^1^ iCell Gene Therapeutics LLC, Research & Development Division, Long Island High Technology Incubator, Stony Brook, NY, USA; ^2^ Department of Pathology, Stony Brook Medicine, Stony Brook, NY, USA; ^3^ Department of Internal Medicine, Stony Brook Medicine, Stony Brook University Medical Center, Stony Brook, NY, USA; ^4^ Macau Institute for Applied Research in Medicine and Health, Macau University of Science and Technology, Macau, China

**Keywords:** NK cells, immunotherapy, T cell malignancies, CD3CAR

## Abstract

Peripheral T-cell lymphomas (PTCLS) comprise a diverse group of difficult to treat, very aggressive non-Hodgkin's lymphomas (NHLS) with poor prognoses and dismal patient outlook. Despite the fact that PTCLs comprise the majority of T-cell malignancies, the standard of care is poorly established. Chimeric antigen receptor (CAR) immunotherapy has shown in B-cell malignancies to be an effective curative option and this extends promise into treating T-cell malignancies. Because PTCLS frequently develop from mature T-cells, CD3 is similarly strongly and uniformly expressed in many PTCL malignancies, with expression specific to the hematological compartment thus making it an attractive target for CAR design. We engineered a robust 3^rd^ generation anti-CD3 CAR construct (CD3CAR) into an NK cell line (NK-92). We found that CD3CAR NK-92 cells specifically and potently lysed diverse CD3^+^ human PTCL primary samples as well as T-cell leukemia cells lines *ex vivo*. Furthermore, CD3CAR NK-92 cells effectively controlled and suppressed Jurkat tumor cell growth *in vivo* and significantly prolonged survival. In this study, we present the CAR directed targeting of a novel target - CD3 using CAR modified NK-92 cells with an emphasis on efficacy, specificity, and potential for new therapeutic approaches that could improve the current standard of care for PTCLs.

## INTRODUCTION

Peripheral T-cell lymphomas (PTCLs) comprise a diverse subset of aggressive, difficult-to-treat hematological malignancies that are derived from mature T-cells [1]. The standard of care for PTCLs is poorly established, with high rates of relapse, low long-term survival and few treatment options for relapsing and refractory disease [1]. Chimeric antigen receptor (CAR) T-cell immunotherapy may offer a potentially curative option, similar to those for B-cell hematological malignancies [2, 3]. However, reports of targeting T-cell malignancies with CARs are rare despite clear clinical need.

The selection and identification of an appropriate surface target antigen is a critical step for the development of a safe and efficacious CAR therapy [4]. The extracellular antigen must be both specific and sensitive for the cancer, with uniform and reliable expression. In addition, the target antigen expression must be specific in order to avoid on target, off tumor effects. CD3 meets these criteria for a majority of mature T-cell lymphomas and a small subset of T-cell acute lymphoblastic leukemia (T-ALL), as these malignancies express CD3 uniformly and reliably, and CD3 expression is limited to the hematologic compartment [5]. However, while the ideal extracellular target protein for CAR therapy is a surface antigen found on 100% of tumor cells at similar robust intensity, real world leukemia and lymphoma scenarios frequently exhibit less than ideal population homogeneity. Importantly, as we will eventually consider CD3 directed CARs for clinical treatment, CD3 expression is restricted to the hematopoietic compartment, limiting on-tumor off-target effects to T-cell aplasia. Additionally, the aplastic bone marrow stem cells can be rescued in the event that T-cell aplasia must be reversed in a clinical emergency. Overall, we hypothesize that CAR therapy directed against CD3 can be used in order to support the current standard of care as a “bridge to transplant” for patients with severe, relapsing or refractory disease. Alternatively, it can serve as a stand-alone therapy for patients with PTCLs with supportive therapy during reversible T-cell depletion and subsequent infusion [6]. However, because normal T cells express CD3, anti-CD3 CAR therapy precludes the use of traditional CAR-modified T-cells.

Since regular T-cells express CD3 extensively, we used a NK cell line (NK-92) as an alternative method to generate CD3-targeted CARs in order to avoid self-targeting. Natural killer (NK) cells are CD3^-^ and have shorter lifespans relative to T-cells. CAR-modified NK cells would be exhausted shortly after destroying cancer cells, with a turnover time of around 7 to 14 days [7]. Their use may eliminate the need for an inducible safety switch [8-10]. However, introduction of an inducible suicide switch may be beneficial should NK therapy persist beyond its life expectancy. Additionally, NK cells mediate anti-tumor effects without the risk of graft-*versus*-host disease (GvHD) and have been validated in CAR applications [7, 11]. NK-92 cells are also CD3^-^ and have been effective in a number of clinical trials targeting solid tumor and hematological malignancies [12-18]. In our proof-of-principle study, we demonstrate that CD3CAR NK-92 cells specifically and robustly eliminated CD3^+^ lymphoma/leukemic cell lines and patient samples *ex vivo*. In addition, CD3CAR NK-92 cells effectively controlled and suppressed CD3^+^ Jurkat tumor growth *in vivo* and significantly prolonged survival. To our knowledge, there are no published studies with CAR-modified NK-92 cells targeting T-cell malignancies and no CAR constructs targeting CD3. The combined use of NK cells with a CD3 target may provide a novel approach with a number of new clinically interesting therapeutic applications.

## RESULTS

### Generation and characterization of CD3CAR construct

**Figure 1:**
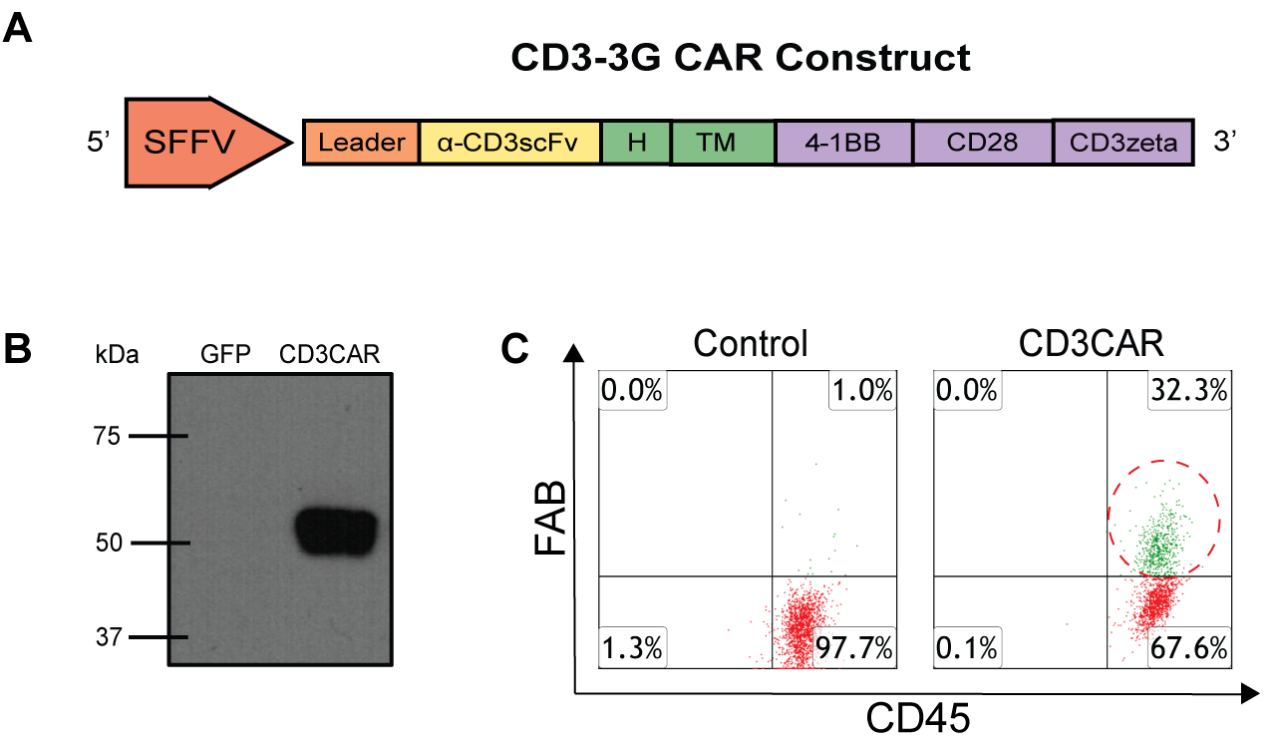
CD3CAR NK-92 functional validation *in vitro* and characterization **A.** Schematic representation of the CD3CAR lentiviral vector (top). The CD3CAR NK-92 construct is a tandem signaling domain that contains: a leader sequence; an anti-CD3scFv; a hinge domain (H); a transmembrane domain (TM); two co-stimulatory domains (CD28 and 4-1BB) that define the construct as a “third generation” CAR^7^; and a CD3zeta intracellular signaling domain. **B.** Western blot analysis of CD3CAR (bottom left). HEK-293FT cells were transduced with lentiviral plasmids for GFP (lane 1) and CD3CAR NK-92 (lane 2) for Western blot analysis at 48h post transduction and probed with mouse anti-human CD3zeta antibody. The expected weight of the CD3CAR NK-92 construct is 58.2 kDa by sequence analysis data (not shown). **C.** Flow cytometry analysis of CD3CAR NK-92 expression on NK-92 cell surface for vector control NK-92 cells and CD3CAR NK-92 cells (bottom right). Population in green delineates the transduced CD3CAR NK-92 cells. Gating done against isotype controls.

CD3CAR's modular design consists of an anti-CD3 single-chain variable fragment (scFv) region, CD8-derived hinge (H) and transmembrane (TM) regions, and tandem CD28 [19] and 4-1BB [20] co-activation domains linked to the CD3ζ signaling domain (Figure 1A). A strong spleen focus forming virus promoter (SFFV) and a CD8 leader sequence were used for efficient expression of the CD3CAR molecule on the NK-92 cell surface. CD3CAR protein was characterized by Western blot of HEK293-FT cells transfected with CD3CAR lentiviral plasmid with appropriate vector control. Additionally, anti-CD3zeta monoclonal antibody immunoblots revealed bands of predicted size for the CD3CAR-CD3zeta fusion protein with no bands observed in vector control (Figure 1B).

### Generation of CD3CAR NK-92 cells

Following fluorescence-activated cell sorting (FACS) to enrich for CD3CAR^+^ NK-92 cells, CD3CAR NK-92 transduction efficiency was determined to be 32.3%, as determined by flow cytometry (Figure 1C). After FACS collection of CD3CAR^high^ cells, we maintained stable CD3CAR expression levels at around 30% on NK-92 cells during expansion of up to 3 or 4 months (data not shown).

### CD3CAR NK-92 cells specifically lyse CD3^+^ T-cell ALL cell lines

**Figure 2:**
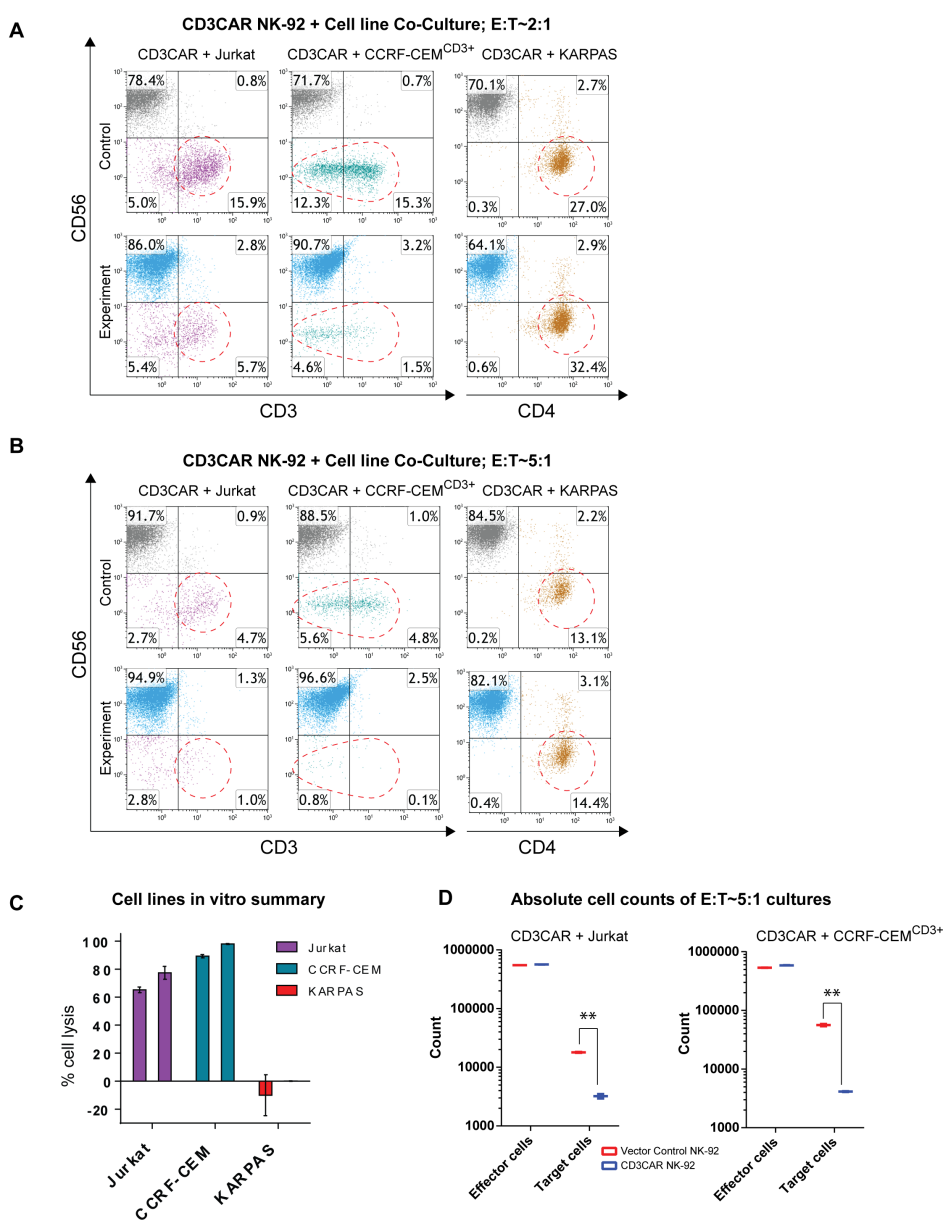
CD3CAR NK-92 cells eliminate CD3-expressing T-ALL cell lines *in vitro* **A.** Co-cultures with CD3CAR NK-92 performed at an effector:target (E:T) ratio of 2:1. T-lymphoblast cell line Jurkat (90% CD3^+^) co-cultured for 6 hours. Sorted CD3^+^ CCRF-CEM (CCRF-CEM^CD3+^) co-cultured for 24 hours. Negative control, CD3^-^ non-Hodgkin's Lymphoma cell line KARPAS 299 co-cultured for 24 hours. Target populations quantified with flow cytometry using CD56 and CD3 to distinguish NK and target cell populations. CD4 used to determine KARPAS population. Populations encircled to highlight lysis amount. **B.** Co-cultures with CD3CAR NK-92 performed at an effector:target (E:T) ratio of 5:1. Experimental conditions identical as 2:1. **C.** Graphical summary of CD3CAR NK-92 *in vitro* assays against T-ALL cell lines. Each bar represents the average % cell lysis for duplicate samples; *N* = 4 experiments for Jurkat and CCRF-CEM^CD3+^ and *N* = 2 experiments for KARPAS. **D.** Absolute cell counts of CCRF-CEM^CD3+^ and Jurkat cultures with CD3CAR NK-92 cells at an E:T ratios of 5:1. Control and CD3CAR treatment samples are labeled in red and blue respectively with effector and target cell counts performed via FACS analysis from Kaluza flow cytometry software (Beckman Coulter).

To assess CD3CAR NK-92 anti-tumor activity, we conducted co-culture assays using Jurkat and CCRF-CEM^CD3+^ T-ALL cell lines. While Jurkat is a CD3^+^ cell line (Supplementary Figure S1), wild-type CCRF-CEM cells only express a small subset of CD3^+^ cells (~20%) with a dominant majority CD3^-^ population center (Supplementary Figure S2). Thus, prior to experiments, CCRF-CEM leukemic cells were enriched for CD3^+high^ (CCRF-CEM^CD3+^) via FACS to test the idea of target specific lysis. Co-culture assays were performed with both wild type and sorted CCRF-CEM cells. We observed that CD3CAR NK-92 cells consistently demonstrated robust lysis of leukemic cells. Following a 6-hour incubation at a low effector to target cell (E:T) ratio of 2:1, CD3CAR NK-92 cells effectively lysed over 60% of Jurkat cells, with close to 80% lysis at an E:T ratio of 5:1 (Figure 2A, 2B, 2C). CCRF-CEM^CD3+^ cells expressed as a smear of CD3 expression after sorting (with evenly distributed CD3 surface density), suggesting that the overall population is CD3^+^ even with disparity in CD3 signal detection (Supplementary Figure S2). After 24 hours of co-culture, CD3CAR NK-92 cells efficiently lysed 85% of CCRF-CEM^CD3+^ cells at an E:T ratio of 2:1, with close to 100% lysis at an E:T ratio of 5:1 (Figure 2A, 2B, 2C), suggesting that CD3CAR NK-92 cells successfully ablated even CD3^dim^ CCRF-CEM^CD3+^ cells. In contrast, when co-culturing CD3CAR NK-92 cells against wild-type CCRF-CEM, a specific cytotoxicity assay (conducted to confirm robustness of assay data, see Materials and Methods) determined around 30-40% lysis of the total CCRF-CEM population, consistent with the lower CD3^+^ phenotype described previously (Supplementary Figure S3). CD3CAR NK-92 cells did not lyse the CD3^-^ lymphoma cell line KARPAS (negative control), highlighting targeting specificity to CD3 expressing cells (Figure 2C).

To determine the possibility of confounding NK cell expansion with killing efficiency due to binding of target antigen, absolute cell counts were performed to verify the significance of lysis. While NK cells have insufficient time to expand during a typical 24 hour experiment (doubling time >48 hrs), absolute counts of residual target cells left in CCRF-CEM and Jurkat co-cultures show statistically significant differences in cell populations due to tumorlysis (Figure 2D). In particular, Jurkat co-cultures show lysis of CD3^+^ cells but insignificant percentage change in the gated CD3^-^ population. CCRF-CEM^CD3+^ co-cultures show lysis action across the entire CCRF-CEM^CD3+^ population due to the fact that CCRF-CEM was sorted for highly expressing CD3^+^ cells which skews the population towards expressing CD3. In addition, 6 hour incubations (*n* = 4) of CD3CAR NK-92 cells with Jurkat show significant lysis compatible with that of 24 hour co-cultures for other cell lines, ruling out any NK-92 expansion influence during that limited time range. Furthermore, we conducted specific cytotoxicity assays where cytotoxicity was measured by comparing the survival of CD3^+^ target cells relative to the survival of a negative control in the same tube cultured with CD3CAR NK-92 cells. This approach was used for both Jurkat and wild-type CCRF-CEM cell lines, showing comparable results with our initial assays (Supplementary Figure S3).

### CD3CAR NK-92 cells specifically target and lyse CD3^+^ populations in primary patient peripheral T-cell lymphoma samples

**Figure 3:**
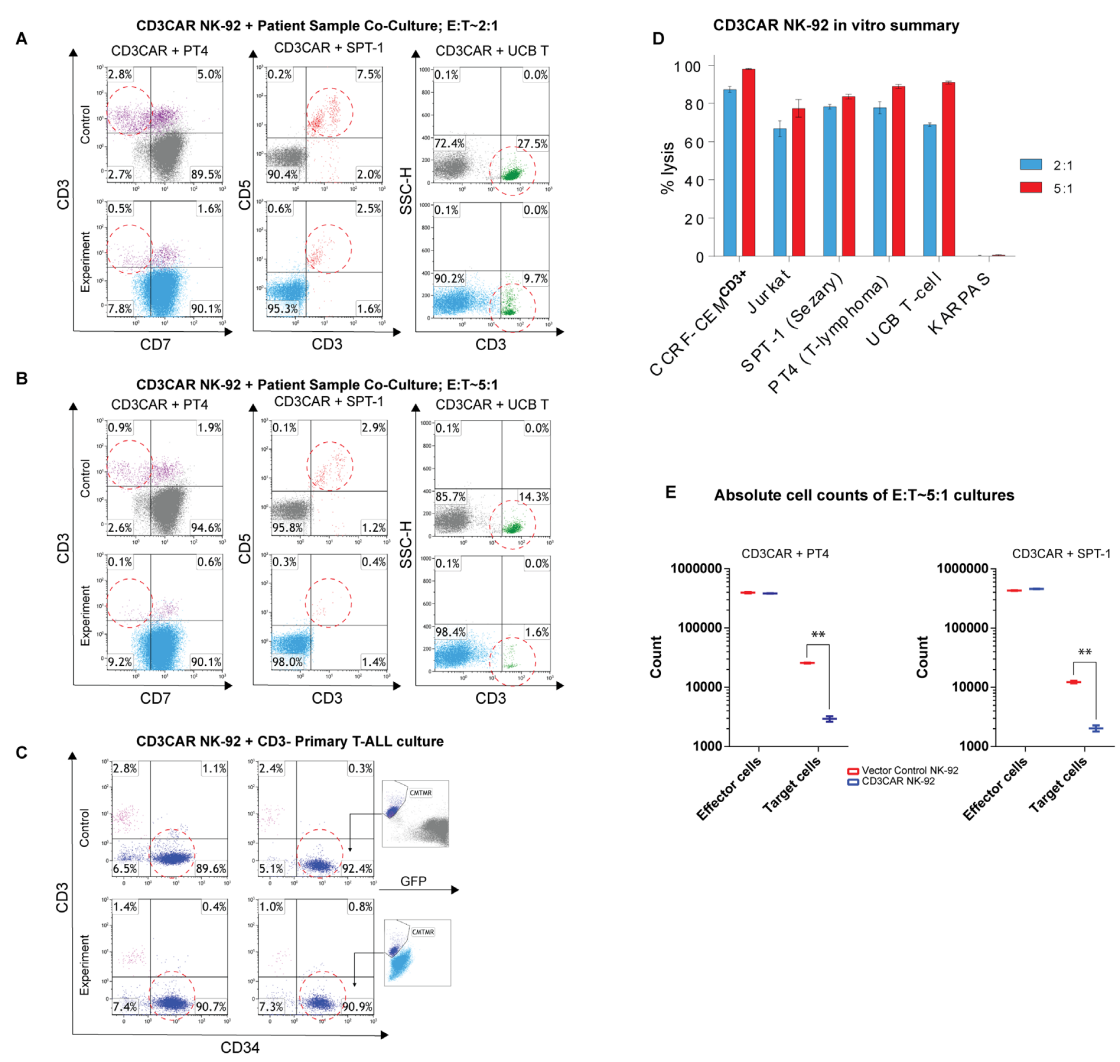
CD3CAR NK-92 cells display robust killing ability for multiple primary CD3+ leukemic cells obtained from patient bone marrow aspirate samples **A.** Co-cultures were carried out at an E:T ratio of 2:1 for 24 hours. Ability of CD3CAR NK-92 cells to lyse target cells, % cell lysis, determined by relative amounts of residual target cells post co-culture. CD3CAR NK-92 cells lyse patient sample PT4 (*N* = 4) (unclassified PTCL phenotype CD7^-^ CD3^+^) and SPT-1 (Sézary Syndrome) (*N* = 2) leukemic cells obtained from patient bone marrow aspirate expressing CD3 as well as normal human T cells (UCB T) isolated from cord blood (*N* = 4). **B.** Co-cultures carried out for 24 hours at an E:T ratio of 5:1. Experimental conditions identical to above. **C.** CD3CAR NK-92 cells co-cultured with T-ALL patient sample expressing majority CD3^-^ tumor burden with a small population of normal T-cells (purple). Co-cultures were conducted in E:T ratios of 2:1 and 5:1 for 24 hours. Cytotracker dye (CMTMR) was used to stain T-ALL sample due to heterogeneity in flow phenotype. **D.** Bar graphs summarizing the cytotoxic activity of CD3CAR NK-92 cells against all types of CD3+ cell populations. T-lymphoblast cell lines and patient samples from T-cell lymphomas expressing CD3 co-cultured with CD3CAR NK-92 cells in the indicated E:T (effector:target) cell ratios of 2:1 and 5:1. % cell lysis values determined using the CD3^+^ total population as the target population with the exception of PT4, where the CD3^+^ CD7^-^ population was designated as the target cell population for enhanced specificity. **E.** Absolute cell counts of selected PT4 and SPT-1 co-cultures at an E:T ratio of 5:1 over 24 hours. Control and CD3CAR treatment samples are labeled in red and blue respectively. Effector and target cell counts were obtained from Kaluza flow cytometry software (Beckman Coulter).

Flow cytometry analysis of SPT-1 (Sézary Syndrome, a classified PTCL) and PT4 (unclassified PTCL) patient samples reveal strong and consistent CD3 expression in population subsets (data not shown). In 24 hour co-culture, CD3CAR NK-92 cells lysed 80% of SPT-1 leukemic cells at a low E:T ratio of 2:1 (Figure 3A). 24 hour co-culture of CD3CAR NK-92 cells with PT4 resulted in >80% ablation of CD3^+^CD7^-^ malignant cells at a low E:T ratio of 2:1 (Figure 3A). At an E:T ratio of 2:1, CD3CAR NK-92 cells also ablated 70% of GFP-transduced normal T cells derived from umbilical cord blood (UCB-T) (Figure 3A). Notably, at an E:T ratio of 5:1, CD3CAR NK-92 fully ablated target SPT-1 and PT4 patient samples (Figure 3B). To further investigate the specificity of the CD3CAR NK-92 cells in targeting primary leukemia patient samples, we considered a T-ALL patient sample expressing only a small subset of CD3^+^ T-cells, with a predominantly CD34^+^ CD3^-^ tumor burden (Figure 3C). The CD3CAR NK-92 cells lysed over 50% of CD3^+^ T-cells at an E:T ratio of 5:1, with no change observed in the CD34^+^ CD3^-^ tumor population (Figure 3C). Similar to above, we extrapolated absolute cell counts to analyze specific cultures and observe significantly lower target cell counts in cultures with CD3CAR NK-92 treatment due to tumorlysis (Figure 3E). Overall, at a low E:T ratio of 2:1, CD3CAR NK cells show significant CD3-specific ablation in patients with heterogeneous CD3^+^ T cell malignancies and T-ALL cell lines, with near 100% lysis of target cells at an increased E:T ratio of 5:1 (Figure 3D).

### A dosage dependent relationship is observed in CD3CAR NK-92 lysis of target cells

**Figure 4:**
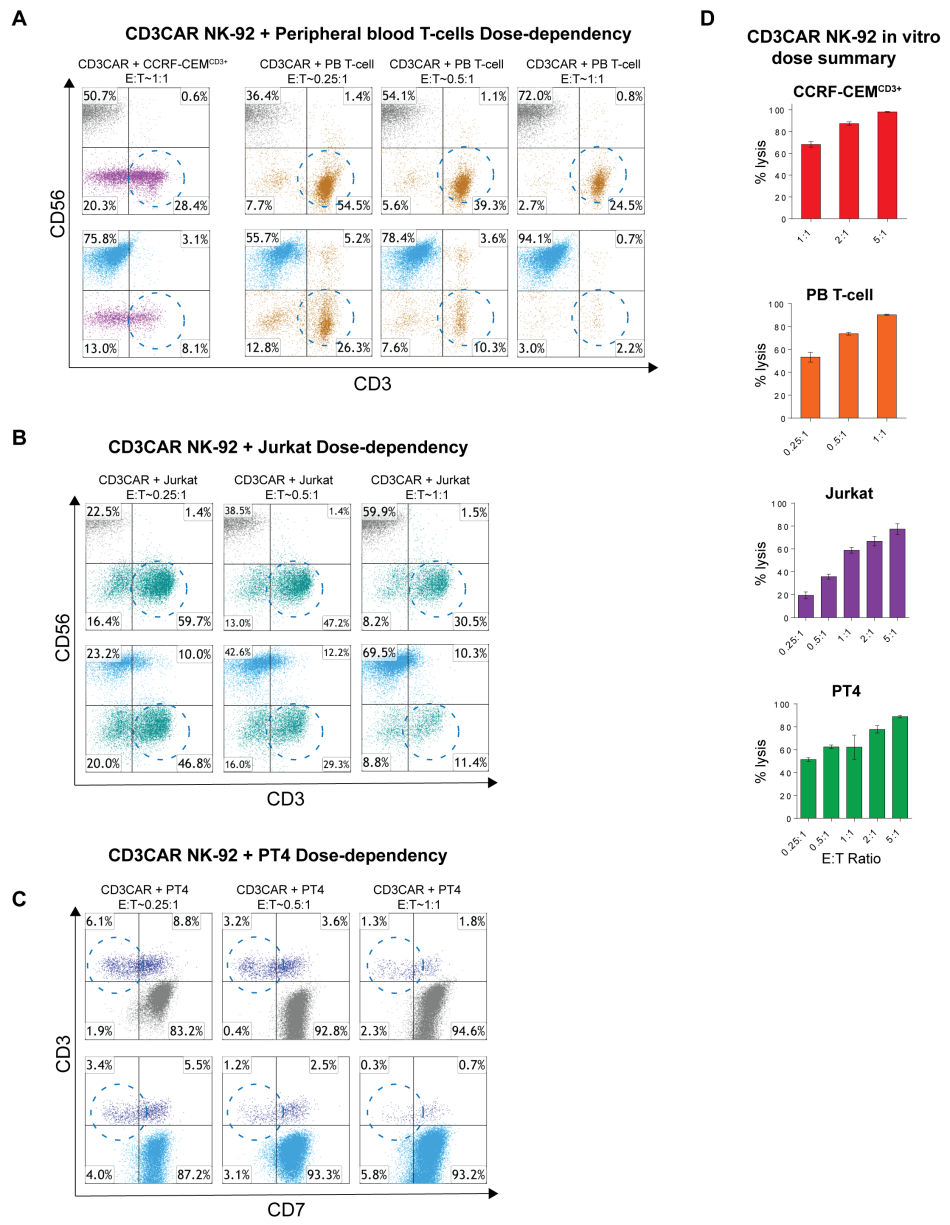
CD3NK-CAR NK-92 dose-dependent lysis of malignant cells **A.** Flow cytometry schemes showing dose-dependent CD3CAR NK-92 co-cultures with CCRF-CEM^CD3+^ and peripheral blood derived (PB) T-cells. E:T ratios were decreased to a lower bound of 0.25:1 (25,000 effector cells to 100,000 target cells) and encircled populations represent lysis effects of interest. Co-cultures were conducted over 24 hours. **B.** Dose-dependent flow analysis of CD3CAR NK-92 cells with Jurkat cell line. Co-cultures were conducted for 6 hours and populations analyzed. **C.** Dose-dependent co-cultures of CD3CAR NK-92 cells with PT4 (unclassified PTCL) primary patient samples obtained from bone-marrow aspirate. **D.** Bar graphs summarizing the dosage relationships between CD3CAR NK-92 dose and target cell depletion. Bars represent the average of duplicate samples with CCRF-CEM^CD3+^ (*N* = 4), Jurkat (*N* = 4), PB T-cell (*N* = 2), and PT4 (*N* = 4).

Notable safety features of NK CAR therapy are a short NK cell half-life and thus transient, defined CD3CAR activity [11]. We investigated CD3CAR NK-92 dose-dependent tumorlysis in T-ALL cell lines and CD3+ leukemic patient samples. All co-culture data was obtained for 24 hour incubation, with the exception of Jurkat cells (6 hours). For CD3CAR NK-92 co-cultures with Jurkat and PT4, the lowest E:T ratio was set to 0.25:1 with an escalating scale up to 5:1. In both experiments, lysis of target cells increased linearly with CD3CAR dosage (Figure 4A, 4B, 4C, 4D). Notably, at a very low E:T ratio of 0.25:1, CD3CAR NK-92 cells already lyse 50% of PT4 cells (Figure 4D). Furthermore, CD3CAR NK-92 co-culture with CCRF-CEM and with peripheral blood (PB) derived T-cells revealed a similar dose-dependent response, with equivalent CD3^+^ cell lysis observed at lower dosages. At an E:T ratio of 1:1, CD3CAR NK-92 cells rapidly depleted 71.5% of CCRF-CEM cells and almost 100% of peripheral blood (PB) derived T-cells at 24 hours (Figure 4A). Combined, these results support the use of CD3CAR NK cell therapy as a therapeutic agent, for instance, to achieve short-term remission for patients with minimal residual disease (MRD) who are to undergo curative hematopoietic stem cell transplantation for PTCLs [9].

### CD3CAR NK-92 cells exhibit significant control and reduction of tumor *in vivo*

CD3CAR NK-92 cells exhibit profound anti-leukemic activity *in vivo*, controlling the growth of Jurkat tumors in a xenogeneic mouse model. Sublethally irradiated NSG (NOD.Cg-*Prkdc^scid^ Il2rg^tm1Wjl^*/SzJ) mice (Jackson Lab, Bar Harbor, ME) were intravenously injected with 1.0 × 10^6^ firefly luciferase-expressing (Luc^+^) CD3^+^ Jurkat cells at day 0, with visible tumor formation using IVIS by day 3 or 4. Mouse injection rationale and approach was based on a projection for maximum efficacy, by administering a one course dose of NK-92 cells during its 7-14 day life expectancy.

**Figure 5:**
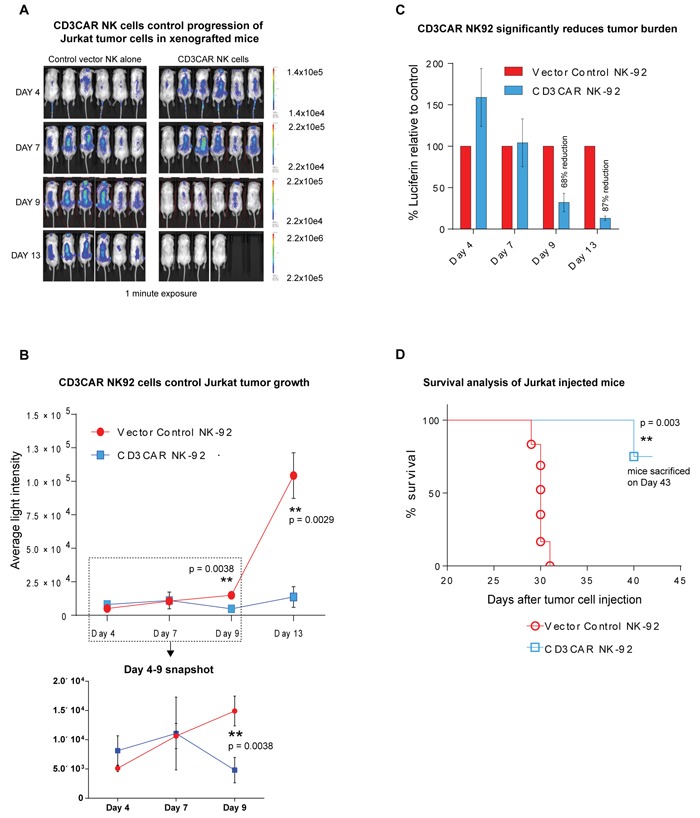
CD3CAR NK-92 cells demonstrate profound anti-leukemic effects *in vivo* **A.** Elimination of luciferase-expressing Jurkat cells in xenografted mice treated with CD3CAR NK-92 as measured via IVIS imaging. NSG mice were sublethally irradiated and, after 24 hours, intravenously injected with 1 × 10^6^ luciferase-expressing Jurkat cells (Day 0) to induce measurable tumor formation. Three days (Day 3) following Jurkat cell injection, mice were intravenously injected via tail vein with one course consisting of a total of 15 × 10^6^ CD3CAR NK-92 cells or vector control NK-92 cells (*N* = 6 per group) during the window of the NK cell life expectancy ending on Day 10. On Day 10, t­­­­wo mice died most likely as a result of stroke from injection procedure and NK cell aggregation. Two additional low dose injections totaling 5 × 10^6^ CD3CAR NK-92 cells were administered through Day 14 and 23 to see if this tumor control could be maintained. On days 4, 7, 9, and 13, mice were injected subcutaneously with RediJect D-Luciferin and subjected to IVIS imaging. The % cell lysis by CD3CAR NK-92 relative to control was determined via luciferin signal. **B.** CD3CAR NK-92 controls Jurkat tumor growth *in vivo*. Average light intensity (in photons per second) measured for the CD3CAR NK-92 injected mice was compared to that of vector control NK-92 injected mice. P-values are indicated at specific time-points, demonstrating a statistically significant reduction in the relative tumor burden by CD3CAR NK-92 cells as compared to vector control. A zoomed in version of the graph from Days 4-9 is included to better illustrate the error measurements and tumor growth. **C.** CD3CAR NK-92 reduces Jurkat tumor burden *in vivo*. Percent luciferin signal measured for CD3CAR NK-92 injected mice and demonstrated as percent difference in signal from vector control NK-92 injected mice. The percent reduction in the tumor burden calculated on days 4, 7, 9, 13, and 17. **D.** CD3CAR NK-92 treated mice survive significantly longer than control. Kaplan-Meier survival curve for CD3CAR NK-92 treated mice compared to vector control treated mice. Log-rank (Mantel-Cox) test p-values as shown and on Day 43, all CD3CAR-treated mice sacrificed for persistency studies. Two mice that died from the injection procedure were excluded from the survival curve and statistics calculation.

To quantitate tumor burden, we measured and compared average light intensity for CD3CAR NK-92 cell injected mice vs that of vector control NK-92 injected mice (Figure 5A and 5B). Three days following Jurkat-Luc+ cell injection (Day 3), mice were intravenously administrated with one course of 15 × 10^6^ CD3CAR NK-92 or vector control NK-92 cells, with 6 mice per group during the window of the NK cell life expectancy and concluding by day 10. By day 9, this led to a 68% tumor reduction and an 87% reduction by day 13 (Figure 5C). On day 10, 2 mice from the CD3CAR treatment group died within 30 minutes after cell infusion, most likely due to stroke from the injection procedure and NK-92 cell aggregation. Therefore, these two mice were excluded from the survival curve and statistics pool. Two additional low dose injections totaling 5 × 10^6^ CD3CAR NK-92 cells were administered on days 14 and 23 to see if this tumor control could be maintained. Consistent with initial injections, tumor burden reduction remained consistent around 87% through Day 23. Future experiments could be designed to determine an optimal dose that is related to maximum efficiency.

In the xenogeneic in *vivo* mouse model, CD3CAR NK-92 cells robustly reduced tumor burden (Figure 5B, 5C) and significantly prolonged survival in Jurkat-injected NSG mice compared to control (Figure 5D). However, CD3CAR NK cells appeared to fail to completely eradicate established human leukemic cells, and leukemic cells ultimately relapsed and retained CD3 expression (data not shown). Relapsed leukemic cells may re-emerge from some leukemic reservoirs, where human CD3CAR NK cells have limited access, or from a lack of human NK cell persistence, as mice may not be able to provide optimal niches for human NK cell homing and persistence. Consistent with this notion, we collected spleens from the earliest dying mice, 17 days after the last CD3CAR NK cell injection, and were unable to detect human CD56+ NK cells by flow cytometry (data not shown).

## DISCUSSION

CAR therapy has emerged as a promising approach for the treatment of various hematological malignancies [2]. To date, current efforts have focused on CAR T-cells that have been effective in addressing various B-cell malignancies using CD19CAR therapy, and such efforts have been validated in clinical trials to significant curative effect [2, 3]. However, peripheral T-cell lymphomas (PTCLs) are aggressive lymphomas with no upfront treatment standard, and severely lack curative regimens. Therefore, CAR NK and T-cells may provide a new avenue for treatment. In our studies, CD3 was selected as the target for CAR-engineered NK cells, since most mature T-cell lymphoma and some leukemia are CD3^+^. Additionally, CD3 is uniformly expressed on the tumor cell populations and its expression is limited to the hematopoietic compartment but absent from hematopoietic stem cells and non-hematologic cells [5]. Furthermore, anti-CD3 monoclonal antibodies (OKT3) have been developed to target CD3 in clinically approved trials for acute organ rejection in patients undergoing cardiac, hepatic, and renal transplants as well as GvHD [21]. Anti-CD3 antibodies have also been used in clinical trials for T-cell lymphomas [22] as well as a variety of autoimmune disorders including recent onset of Type 1 diabetes and inflammatory bowel diseases [23, 24].

On the basis of CD3 as a clinically validated target, our approach using CAR-modified NK cells represents a complementary therapy approach to CAR T-cells with an emphasis on the short life span and cytotoxic effector mechanisms that are distinct from CAR T-cells [7]. CAR NK cells are capable of targeting and lysing tumor cells through the chimeric antigen receptor mechanism. In addition, NK cells express the IgG Fc fragment, low affinity III receptor (FcRYIII) that enable them to mediate antibody-dependent cell-mediated cytotoxicity [25]. These factors allow NK cells to possess a less pro-inflammatory profile while simultaneously manifesting effector functions via multiple mechanisms. Several pre-clinical studies for CAR NK cells or NK-92 cells have been conducted, and CAR targeting antigens have included CD19, CD20, CD244 ganglioside GD2, CD138, CS1, GPA7, and HER2 [7]. Recently, two clinical studies of CAR-modified NK cells are underway (NCT00995137 and NTC01974479).

The potential disadvantage of using NK cells in CAR therapy is a lack of persistency and difficulty in acquiring high efficiency transduction that may reduce long-term efficacy [7]. This may be ameliorated through using CD3CAR T-cells. However, as CD3 is also expressed in CAR T-cells, this offsets their ability to target the CD3 antigen and may induce fratricide. Thus, engineering CD3CAR T-cells require inactivation of the endogenous CD3 antigen in T-cells using genomic editing techniques as a precursor to building armored CARs. In addition, while the use of CD3CAR on primary NK cells remain a compelling direction, and could be ultimately used for clinical treatment with similar efficacy, there are cell-culture concerns with autologous NK cell expansion [7]. High yield isolation of primary NK cells remain difficult and cell expansion is limited in *vitro* [7]. Furthermore, primary NK cells are difficult to transduce with high efficiency, necessitating the need for specific engineering optimization. However, future experiments could be designed to test the viability and efficacy of CD3CAR primary NK cells.

The treatment of CD3CAR NK-92 cells likely results in “on-target” toxicities, as observed in other CARs, such as CD19CAR therapy which is associated with B-cell aplasia, albeit reversible, and prolonged aplasia is generally not seen in clinical trials [11]. The resultant T-cell aplasia from CD3CAR NK-92 treatment may be of greater concern. In particular since normal T-cells express CD3 in a strong and consistent manner and usually at a higher intensity than tumor cells, such aplasia will be almost a certainty. We show in our in *vitro* studies that the CD3CAR NK-92 cells achieve significant lysis of regular T-cells (Figure 3) comparable to those of CD3^+^ tumor cells from leukemia cell lines and patient samples. Therefore, it is necessary for CD3CAR NK-92 cell activity to be strictly transient. For NK cells, which have life-spans of only around 2 weeks, transient activity is expected and NK-92 cell infusions have achieved relatively few cases of minor GvHD or cytokine storm in clinical studies [7, 25]. For further consideration in the clinic, transient CD3CAR expression could overcome on-target toxicities through the use of mRNA techniques or the incorporation of a safety switch or inducible suicide gene.

In our preclinical study, we demonstrate that the CD3CAR NK-92 cells have the potential to be an effective treatment option for aggressive and difficult to treat PTCLs with the benefit of short-term, reversible T cell depletion [11]. We show evidence that CD3CAR NK-92 cells specifically and potently eliminate CD3^+^ T-cell leukemic cell lines and lymphoma patient samples in a proof-of-concept demonstration for allogenic or autologous NK cell-based CAR therapy. One important factor for in *vitro* studies, however, is the NK intrinsic cell-lytic ability’ to lyse tumor cells. We show in specific cytotoxicity assays (as a corollary to the co-cultures) that while the overall CD3CAR lysis of the Jurkat cell line is consistent with our initial assays, wild-type NK-92 cells lyse around 35% of Jurkat cells when E:T ratios are increased to 5:1 (Supplementary Figure S3). This intrinsic lytic ability accounts for the mismatched ratios of the in *vitro* assays in the vector control treatments, however, it is negligible at lower E:T ratios (Supplementary Figure S3). As Jurkat cells tend to be Fas^+^, the lysis of Jurkat within a relatively rapid timeframe might be due to the cell-specific susceptibility to NK-mediated cell death as a result of Fas ligand binding and subsequent apoptotic activation [26]. Furthermore, prior reports have also established that Jurkat cells are also susceptible to tumor necrosis factor-related apoptosis-inducing ligand (TRAIL) binding which NK cells can also activate [26]. We then demonstrate the CD3CAR NK-92 cell activity is limited to the CD3^+^ cells only when gating for Jurkat CD3^+^ depletion. In addition, specific cytotoxicity assays on wild-type CCRF-CEM which expresses a small subset of CD3^+^ cells (~20%), show a correspondingly lower CD3CAR NK-92 lytic activity of around 30-40% cell lysis when compared to lysis of sorted CCRF-CEM^CD3+^ (Supplementary Figure S3). These findings support the specific ability of the CD3CAR NK-92 cells to target the CD3 antigen without off-target effects.

In the in *vivo* xenogeneic mouse model, CD3CAR NK-92 cells exhibited remarkable efficacy with an 87% reduction in tumor burden when compared to control mice (Figure 5) during the NK cell turnover window of approximately two weeks. We find that, in general, sustained dosing of mice after the turnover window only promotes maintenance of leukemic suppression of around 80% and leukemic recovery occurs after injections were halted (data not shown). By day 22, some Jurkat leukemic cells can be seen reemerging from niches within the mice. The results suggest at both an incomplete eradication of leukemia by CD3CAR NK-92 cells and the likeliness of murine niches that remain inaccessible to human NK cells as seen in other xenograft models [16, 27-29]. In particular, this suggests that NK cell therapy is transient and prone to NK cell turnover without repeated dosing.

We posit, therefore, that CD3CAR NK therapy can potentially be used in the treatment of residual T cell malignancies after the exhaustion of current therapeutic options to allow relapsing and refractory patients to qualify for curative hematopoietic stem cell transplant. As reported for general NK cell therapy, we suggest that CD3CAR NK-92 cells can be administered as a quick tumor-lytic regimen followed by relatively quick cell turnover [25]. Administration of CD3CAR NK cells could eliminate leukemia cells in a short time period, followed by bone-marrow transplant (BMT) to rescue lymphopenia. Alternatively, a low dose of donor T cells may be also used to support transient lymphopenia. In this application, since BMT necessitates the elimination of residual leukemia cells or self-reactive T-cells, CD3CAR treatment can be used to promote the transient ablation of target cells, thus qualifying new patients for supportive and continuing therapy.

Furthermore, graft-*versus*-host disease (GvHD) remains a source of significant morbidity and mortality in the setting of allogeneic stem cell transplantation (SCT), and minimal residual disease (MRD) level is an essential independent predictor of disease relapse [30]. The transient persistence of CD3CAR NK cells during and after the infusion of bone marrow stem cells could result in significant *in vivo* depletion of donor T lymphocytes responsible for GvHD and the donor anti-leukemic effect. In this setting of allogeneic SCT for CD3^+^ T cell malignancies, CD3CAR NK cells may serve a dual function of reducing the risk of GVHD and eliminating MRD. CD3CAR NK technology could also further be expanded to reduce the incidence and severity of acute organ rejection with reversible depletion of host T-cells during transplant.

We have shown thus far that CD3CAR transduced NK-92 cells have potent lysis effects in *vitro* with specific dose-dependent relationships that can be extended into the clinic as well as significant effects *in vivo* that show improved survival in CD3CAR treated mice. Our preclinical study may be useful for projecting the viability of using CD3CAR modified NK cells for the treatment of CD3^+^ malignancies and as a bridge to bone-marrow transplant or other current standard of care practices.

## MATERIALS AND METHODS

### Primary tumor cells and cell lines

Human primary tumor samples were obtained from residual samples following a protocol approved by the Institutional Review Board of Stony Brook University. KARPAS 299, CCRF-CEM, Jurkat, and NK-92 cell lines were obtained from ATCC (Manassas, VA). NK-92 cells were cultured in filtered NK-92 cell media (defined as alpha-MEM without ribonucleosides and deoxyribonucleosides with 2mM L-glutamine, 1.5 g/L sodium bicarbonate, 12.5% heat-inactivated horse serum, 12.5% heat-inactivated FBS, 1X Pen/Strep, 0.2% inositol, 0.02% folic acid, and 50 uM beta-mercaptoethanol, supplemented with IL-2 (300 IU/mL), unless otherwise specified. KARPAS 299, CCRF-CEM, and Jurkat cell lines were cultured in RPMI, 10% FBS, 1x Pen/Strep (Gibco, Waltham, MA, USA).

### CAR construct design and lentiviral transduction

The CD3-specific CAR (pRSC.SFFV.CD3.3G) consists of an extracellular single-chain variable fragment (ScFv) domain directed against the target protein CD3, and an intracellular tandem signaling domain comprised of a CD28 and 4-1BB coactivation domains upstream of a CD3ζ activation domain. The three signaling domains define the construct as a third generation CAR. Viral supernatant containing the CD3-CAR was produced by 293FT cells co-transfected with pMD2G and pSPAX viral packaging plasmids containing either pRSC.SFFV.CD3.3G or GFP vector control using Lipofectamine 2000 (Life Technologies, Carlsbad, CA) as per manufacturer's protocol. NK-92 cells were then cultured and transduced (see detailed lentiviral production). For transduction, NK-92 cells were incubated in BSA-blocked 6 well plates with lentiviral supernatant containing the CD3CAR or vector control construct overnight.

### CAR detection on transduced NK-92 cells

NK-92 cells were washed and suspended in FACs buffer (0.2% BSA in DPBS) 3 days after transduction. Labeling with antibodies was performed as follows. Normal goat IgG (Jackson Immunoresearch, West Grove, PA) was used to block nonspecific binding. Each NK-92 cell sample was probed with Biotin-labeled polyclonal goat anti-mouse F(Ab’)^2^ (1:250, Jackson Immunoresearch, West Grove, PA) for 30 minutes at 4°C. Cells were washed once, and resuspended in FACs buffer. Cells were then stained with PE-labeled streptavidin (1:250, Jackson Immuno Research, West Grove, PA) for 30 minutes at 4°C. Cells were washed with FACs buffer, and resuspended in 2% formalin. Flow cytometry was performed using a FACS Calibur instrument (Becton Dickinson, Franklin Lakes, NJ), and results were analyzed using Kaluza software (Beckman Coulter, Brea, CA).

CD3CAR^+^ NK cells were sorted via FACS for the experiments. These sorted cells maintained stable expression when cultured over at least 3 or 4 months, as determined by transduction efficiency. Additionally, sorted control NK and CD3CAR NK cells had similar growth curves over time.

### Co-culture assays and gating schemes

CD3CAR and vector control NK-92 cells were incubated with CD3 expressing T-ALL cell lines: Jurkat (*n* = 4) and CCRF-CEM (*n* = 4), in addition to primary patient cells: CD3+ umbilical cord blood (UCB) (*n* = 4) or peripheral blood (PB) (*n* = 2) derived T-cells, and CD3 expressing primary human leukemia cells: SPT-1 (adult Sézary syndrome) (*n* = 2) and PT4 (unclassified PTCL) (*n* = 4). For negative controls, CD3CAR and vector NK-92 cells were incubated with CD3 negative non-Hodgkin's Lymphoma cell line KARPAS 299 (*n* = 2) and a T-ALL patient (T-ALL 1) (*n* = 2) whose majority tumor burden was CD3 negative.

Prior to co-culture assays, wild-type CCRF-CEM cells were sorted by FACS for CD3^+^ expression. As only a small subset of wild-type CCRF-CEM cells expressed CD3, the highest CD3 expressing cells were gated and collected and expanded in *vitro*. Flow cytometry further confirmed the population shift as a result of sorting, with over 50% of the sorted CCRF-CEM^CD3+^ population expressing CD3 as a smear compared to ~20% dim expression for wild-type CCRF-CEM (Supplementary Figure S2).

Co-cultures were carried out at ratios of 2:1 and 5:1 (200,000 and 500,000 effector cells to 100,000 target cells respectively) in 1 ml of 2.5% serum NK-92-cell culture media without IL-2. Dosage dependent experiments were performed at lower ratios from 0.25:1 (25,000 effector cells to 100,000 target cells), to 0.5:1 to 1:1. After 24 hours, remaining live cells were harvested and stained with mouse anti-human CD56 and CD3 antibodies for cell line and T-cell depletion assays. Mouse anti-human CD5, CD7, and CD34 were additionally combined to stain SPT-1, PT4, and T-ALL 1 patient samples. CD56 single positives denote NK-92 cells, and CD3 generally denotes the target cell populations. For SPT-1, the general phenotype is CD3^+^ thus gating with CD56 and CD3 separates out the populations. PT4 is an unclassified PTCL in which the malignant cells are CD3^+^, CD7^-^. T-ALL 1 is a leukemia that is CD5^+^, CD34^+^, and CD3^-^. All cells were washed with FACS buffer, resuspended in 2% formalin, and analyzed by flow cytometry.

Analysis of anti-leukemic activity was performed by comparing the residual amount of cells left in the appropriate gates in the CD3CAR NK-92 treated samples with the vector control NK-92 treated sample. Analysis was performed using Kaluza Software package (Beckman Coulter), and generation of bar graphs was done in GraphPad Prism. Error bars denote standard deviation in the sample results.

### Specific cytotoxicity assay

Cytotoxicity assays were conducted as described [31] and were performed to verify the co-culture assays above and to provide data robustness. Briefly, cytotoxicity was measured by comparing the survival of CD3^+^ target cells relative to the survival of CD3^-^ negative control cells. These two cell types were combined in the same treatment wells with either CD3CAR transduced NK-92 cells or wild-type NK-92 cells (to measure intrinsic basal line NK-92 cell lytic ability). CD3^+^ target cells were Jurkat (~90% CD3^+^) and wild-type CCRF-CEM (~15% CD3^+^) and were stained with CFSE (carboxyfluorescein diacetate succinimidyl ester). CD3^-^ negative control cells were KARPAs stained with CMTMR (5-(and-6)-(((4-chloromethyl)benzoyl)amino) tetramethylrhodamine) (ThermoFisher). Co-cultures were setup in duplicate wells at 1:1, 2:1, and 5:1 E:T ratios with a 1:1 mix of target cells to non-target cells comprising the total target cell population. Cultures were incubated for 5 hours (Jurkat) or 12 hours (wild-type CCRF-CEM) and 7-AAD (7-aminoactinomycin D; BD) was added to track cell membrane integrity. Flow cytometry acquisition was then immediately conducted. The survival percentage of CD3^+^ CFSE^+^ target cells was calculated by dividing the percentage of live CD3^+^ CFSE^+^ target cells by the percentage of live CD3^-^ CMTMR^+^ negative control KARPAs cells. The corrected survival percentage was then calculated by dividing the survival percentage of CD3^+^ CFSE^+^ target cells by the ratio of live target cells to live non-target negative control cells in a 1:1 control well with no effector CD3CAR cells added. This value serves to normalize and correct for starting cell number variation and spontaneous cell death.

### Xenogeneic mouse model

Male 12-week-old NSG mice (NOD.Cg-Prkdcsid Il2rgtm1Wjl/SzJ) were purchased from the Jackson Laboratory (Bar Harbor, ME) and used under a Stony Brook University IACUC-approved protocol. NSG mice were irradiated with a sublethal (2.0 Gy) dose of gamma irradiation. Next day, mice were intravenously (IV) injected with 1.0 x10^6^ Jurkat cells that had been stably transduced to express luciferase, in order to cause a measurable IV tumor to form. Three days (Day 3) following Jurkat cell injection, mice were intravenously injected via tail vein with one course consisting of 15 × 10^6^ CD3CAR NK-92 cells or vector control NK-92 cells (*N* = 6 per group) during the window of the NK cell life expectancy and concluding by Day 10. Upon injection of NK-92 cells on Day 10, 2 mice subsequently died within 30 minutes after injection procedure. Two additional low dose injections totaling 5 x10^6^ CD3CAR NK-92 cells was administered through Day 14 and 23 to see if this tumor control could be maintained. On days 4, 7, 9, and 13, mice were injected intraperitoneally (IP) with 100 μL RediJect D-Luciferin (Perkin Elmer, Waltham, MA) and subjected to IVIS imaging (PerkinElmer, Waltham, MA). Images were analyzed using Caliper Life Sciences software (PerkinElmer, Waltham, MA).

### Statistics

Xenogeneic model sample sizes were estimated using 2-sample, 2-sided equality power analysis (90% power and <5% significance) without blinding. Unpaired student t-tests were used to determine significance of tumor size area and light intensity. Survival curves were constructed using the Kaplan-Meier method and statistical analyses of survival was performed using a log-rank (Mantel-Cox) test with *P* < 0.05 considered significant. Statistical analyses were performed using GraphPad Prism 6 software. Variance was determined to be similar between the treatment and control group prior to unpaired student *t*-tests.

### Detailed lentivirus production and transduction of NK-92 cells

To produce viral supernatant, 293FT-cells were co-transfected with pMD2G and pSPAX viral packaging plasmids containing either pRSC.SFFV.CD3.3G or GFP lentiviral vector control, using Lipofectamine 2000 (Life Technologies, Carlsbad, CA) according to the manufacturer's protocol, and incubated for 6 hours. Cells were then washed and suspended in DMEM with 10% FBS, sodium butyrate, sodium pyruvate, and HEPES (20mM) (all Gibco, Waltham, MA, USA). Viral supernatant was collected 24 and 48 hours after transfection, cleared of cellular debris via centrifugation and filtration (0.45 uM), aliquoted, and flash frozen in liquid nitrogen for storage at −80°C.

To confirm virus production, 293-FT cells were harvested 48 hours after transfection, lysed in 1 mL RIPA buffer with deoxycholate and protease inhibitor cocktail [32], and 10 uL sample was electrophoresed on a 10% PAGE-SDS gel, and transferred to Immobilon FL (0.45 uM) membrane using the wet cell method. Milk (5%) in TBS/Tween was used to block blots. Blots were probed with anti-CD247/CD3z (Thermo Fisher Holtsvile, NY) at 1:500 overnight, washed 4 times with TBS/Tween, and probed with anti-goat IgG, HRP-conjugated antibody (Thermo Fisher) at 1:5000 for 2 hours. Following additional washes, HRP substrate (HyGlow, Denville, Holliston, MA) was added to the membrane and the membrane was exposed to autoradiographic film.

NK-92 cells (ATCC; Manassas, VA) were cultured for 2 days in the presence of 300 IU/mL IL-2 (Peprotech, Rocky Hill, NJ). A non-tissue culture treated 6-well plate was coated with RetroNectin (Clontech [Takara Bio], Mountain View, CA) at 15 ug/mL in DPBS for 2 hours at room temperature or overnight at 4 °C. Wells were blocked with 2% BSA in PBS for 30 minutes at room temperature, then washed once with PBS. Viral supernatant (CD3CAR or GFP vector control lentivirus) was diluted 1:1 with DMEM containing 10% FBS and added to the washed wells by centrifugation at 2000 g for 2 hours at 32 °C. Wells were washed once with NK-92 cell media, and NK-92 cells were added, 4 mL per well at 0.5 × 10^6^ cells/mL, with IL-2 (300 IU/mL. Plates were centrifuged at 1000 g for 10 minutes and incubated overnight at 37 °C in the presence of 5% CO_2_. The following morning, a second transduction, identical to the first, was carried out. The morning after that, cells were transferred to a fresh non-coated 6-well plate in NK-92 cell media with IL-2 (300 IU/mL), cells were sorted for CD3CAR^+^ NK-92 cells, and subsequently incubated as above for a total of 7 days from transduction.

## SUPPLEMENTARY MATERIAL FIGURES AND TABLE


