# Supplementary material for: Novel anti-CD3 chimeric antigen receptor targeting of aggressive T cell malignancies
Source: Oncotarget. 2016 Aug 2;7(35):56219–32. doi: 10.18632/oncotarget.11019 (PMC5302909; doi:10.18632/oncotarget.11019)
Supplement: Supplementary file 1 [file oncotarget-07-56219-s001.pdf]

Novel anti-CD3 chimeric antigen receptor targeting of aggressive T cell malignancies

Supplementary Material

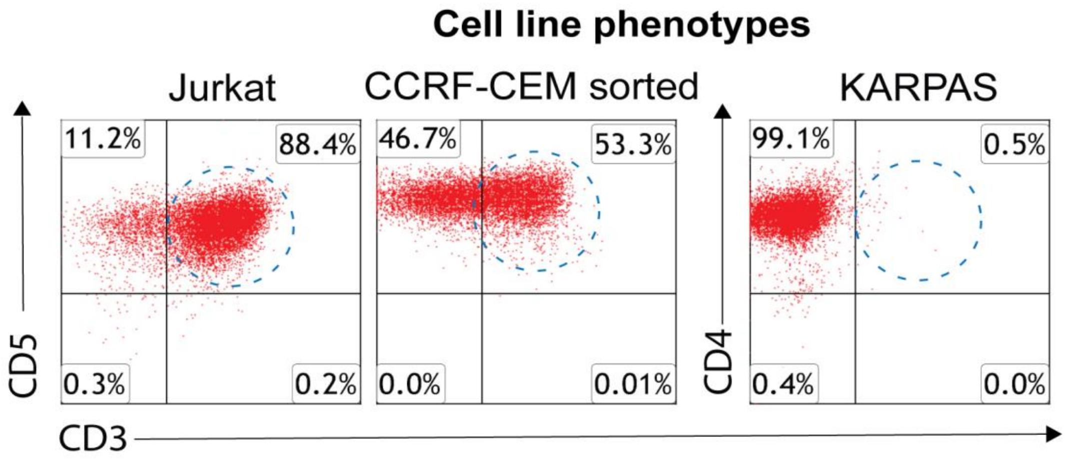

Figure S1: Cell line phenotypes

Flow cytometry analysis of T-cell leukemia cell lines Jurkat, sorted CCRF-CEM^{CD3+}, and KARPAS. Target cell Jurkat is a CD3^{+}CD5^{+} cell line while CCRF-CEM^{CD3+} was sorted for CD3^{high} cells leading a smeared phenotype of gradient CD3 expression when compared to wild-type CCRF-CEM cells. KARPAS is a CD3^{-} negative control cell line that expresses CD4.

Sorted vs. wild-type CCRF-CEM

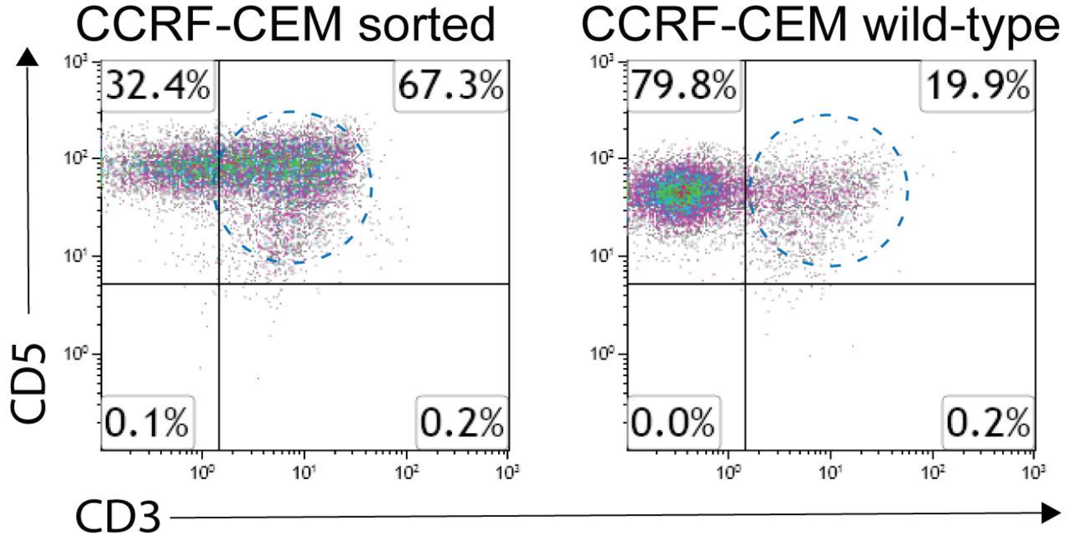

Figure S2: Comparison of wild-type CCRF-CEM with sorted CCRF-CEM^{CD3+}

Flow cytometry density plots comparing wild-type CCRF-CEM cells with sorted CCRF-CEM^{CD3+} cells. The population distribution shows that in wild-type CCRF-CEM populations, the majority of the cells are CD3^{-} and exists as a solid population. The CD3^{+} residuals of wild-type CCRF-CEM exists as a minority population of around 20%. After FACS sorting of this minor population, the resultant CCRF-CEM^{CD3+} population exhibits as a consistent distributed smear likely signifying an overall CD3^{+} population with gradient expression. CCRF-CEM cells are also CD5^{+}.

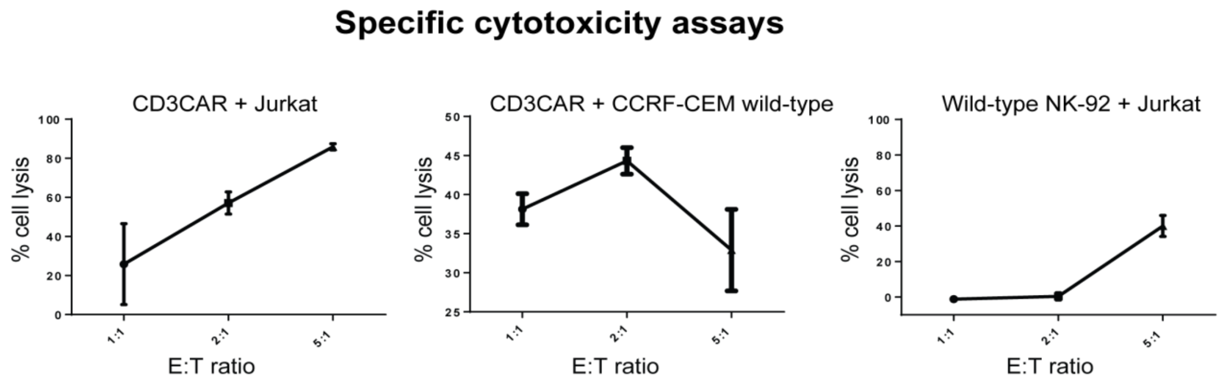

Figure S3: Specific cytotoxicity assays

Specific assays were conducted as described in Materials and Methods and were used as a corollary to support the co-culture assays done in Figures 2 and 3. Briefly, target Jurkat and wild-type CCRF-CEM cells were labeled with CFSE dye and non-target negative control KARPAS were labeled with CMTMR dye. Target and non-target cells were incubated together at 1:1 ratios with varying amounts of CD3CAR or wild-type NK-92 cells to specific E:T ratios. Cytotoxicity was measured by normalizing the relative survival of the target cells against non-target cells by a control sample with no effector cells added. The Jurkat cytotoxicity assay results in % cell lysis numbers comparable with co-culture assays done in Figure 2. Against minority $CD3^+$ wild-type CCRF-CEM, % lysis is correspondingly lower, with saturation reached at around 35% lysis (as predicted by wild-type CCRF-CEM phenotype). The cytotoxicity assays also reveal baseline wild-type NK-92 cell lytic ability against the Jurkat cell line, with negligible non-specific lysis at E:T ratios of 1:1 and 2:1. Upon increasing the E:T ratio to 5:1, this non-specific intrinsic NK-mediated cell killing rose to 35-40%.

NAME	VENDOR	HOST SPECIES	CATALOG #	CLONE #	DILUTION	USE
CD3zeta-CD247	Thermo Fisher	Mouse	MA5-15608	4B10	1:500	Western Blot
Goat anti-mouse HRP	Abnova	Goat	PAB10746	n/a	1:2000	Western Blot
Goat anti-mouse F(AB')[2], biotin	Jackson	Goat	115-066-072	n/a	1:250	F(Ab')2 detection/Flow cytometry
R-Phycoerythrin Streptavidin	Jackson	n/a	016-110-084	n/a	1:250	Secondary for F(Ab')2 detection/Flow cytometry
Anti-human CD3-PerCP	Tonbo Biosciences	Mouse	65-0037-T025	OKT3	1:50	Flow cytometry
Anti-human CD56-PE	Tonbo Biosciences	Mouse	50-0564-T100	NCAM, MY31	1:50	Flow cytometry
Anti-human CD7-APC	Tonbo Biosciences	Mouse	561604	M-T701	1:50	Flow cytometry
Anti-human CD34-PE.cy7	Tonbo Biosciences	Mouse	560710	581	1:50	Flow cytometry
Anti-human CD5-APC	Tonbo Biosciences	Mouse	20-0059-T100	UCHT2	1:50	Flow cytometry
CMTMR	Life Technologies	n/a	C2927	n/a	5 µM	Flow cytometry

Supplementary Table 1: Flow cytometry, Western Blot reagents
